# Results from Canton Grisons of Switzerland suggest repetitive testing reduces SARS-CoV-2 incidence (February–March 2021)

**DOI:** 10.1038/s41598-022-23986-0

**Published:** 2022-11-14

**Authors:** Hossein Gorji, Ivan Lunati, Fabian Rudolf, Beatriz Vidondo, Wolf-Dietrich Hardt, Patrick Jenny, Doortje Engel, Jörg Schneider, Marina Jamnicki, Rudolf Leuthold, Lorenz Risch, Martin Risch, Martin Bühler, Adrian Sommer, Alexa Caduff

**Affiliations:** 1 Laboratory of Multiscale Studies in Building Physics, Empa, Dübendorf, Switzerland; 2 D-BSSE ETH Zürich, Swiss Institute of Bioinformatics, Basel, Switzerland; 3 Federal Office of Public Health FOPH, 3097 Bern, Switzerland; 4 Department of Clinical Research and Veterinary Public Health, University of Bern, Bern, Switzerland; 5 Institute of Microbiology, D-BIOL, ETH Zürich, Zurich, Switzerland; 6 Department of Mechanical and Process Engineering, ETH Zürich, Zurich, Switzerland; 7 Department of Justice, Security and Health, Canton Grisons, Switzerland; 8 Clinical Microbiology, Labormedizinisches Zentrum Dr. Risch, 9470 Buchs, SG Switzerland; 9 Faculty of Medical Sciences, Private University of the Principality of Liechtenstein, 9495 Triesen, Liechtenstein

**Keywords:** Viral infection, Data processing

## Abstract

In February 2021, in response to emergence of more transmissible SARS-CoV-2 virus variants, the Canton Grisons launched a unique RNA mass testing program targeting the labour force in local businesses. Employees were offered weekly tests free of charge and on a voluntary basis. If tested positive, they were required to self-isolate for ten days and their contacts were subjected to daily testing at work. Thereby, the quarantine of contact persons could be waved.Here, we evaluate the effects of the testing program on the tested cohorts. We examined 121,364 test results from 27,514 participants during February–March 2021. By distinguishing different cohorts of employees, we observe a noticeable decrease in the test positivity rate and a statistically significant reduction in the associated incidence rate over the considered period. The reduction in the latter ranges between 18 and 50%. The variability is partly explained by different exposures to exogenous infection sources (e.g., contacts with visiting tourists or cross-border commuters). Our analysis provides the first empirical evidence that applying repetitive mass testing to a real population over an extended period of time can prevent spread of COVID-19 pandemic. However, to overcome logistic, uptake, and adherence challenges it is important that the program is carefully designed and that disease incursion from the population outside of the program is considered and controlled.

## Introduction

### State of knowledge in mass testing

Repetitive testing of people without noticeable symptoms has been proposed as a public health measure in response to COVID-19 pandemic. The concept relies on reducing the effective infectiousness period by isolating the positively tested individuals when they are presymptomatic or asymptomatic. Theoretical studies have demonstrated that repetitive mass testing helps contain the virus spread^1–4^. They suggest that this strategy can contribute to control the local epidemics and might even provide an alternative to extreme interventions with higher social, psychological, or economic costs, such as blanket lockdowns. However, due to logistic challenges and high costs, skepticism still remains against repetitive testing as its benefits haven’t been proven broadly yet^5,6^.

In the current pandemic, several types of SARS-CoV-2 tests with acceptable sensitivity for symptomatic cases have proven successful as diagnostic tools^7^. For repetitive population screening, however, specificity, test-to-notice time, and sensitivity to asymptomatic virus carriers are crucial, as they may hinder the effectivity of mass testing programs, if not chosen properly^5^. In addition, the behavior of the population may jeopardize the positive effects of testing if, for instance, participants do not correctly comply with instructions (i.e. not consistently isolating if tested positive)^5,6^. Finally, the success of mass testing depends on the possibility to cover a sufficiently large fraction of the population and their contacts. More empirical evidence is needed to evaluate the benefits of mass testing when applied to a real population over an extended period of time. There is still lack of field studies of sufficient coverage demonstrating that logistic, uptake and adherence challenges can be overcome.

To date, population-based mass testing has been carried out and documented in countries such as the UK, Denmark, China, South Korea, Austria, Luxembourg, and Slovakia, that have mostly used rapid antigen tests^8–14^. In Slovakia, a few rounds of population-wide mass testing were estimated to yield a 70% decline of infection prevalence^10,11^. Pilot mass testing based on Lateral Flow Devices (LFDs) has been conducted in Liverpool, where lower uptake compared to Slovakia’s was found (only 25% of the population was tested in a 4-week period)^12,13^. Here we report new evidence from a program in the Canton Grisons (Switzerland) suggesting that repetitive virus RNA mass testing is effective in reducing SARS-CoV-2 incidence. Our observational study gives further insight into effectivity of mass testing in presence of a significant number of exogenous contacts resulting from tourists and commuting labor force.

### Test concept in Canton Grisons

The Canton of the Grisons, the largest in surface and easternmost Canton of Switzerland, shares borders with Italy, Austria and Liechtenstein, and has approximately 200,000 inhabitants. A large share of its economy is dedicated to tourism, e.g. in the past winter season, the Canton Grisons received around 200,000 tourists with 20% from abroad. Due to a high proportion of daily to weekly border crossing employees from the neighbouring countries, border disease control measures are less restrictive than in other parts of the world (in particular after the relaxation of the initial lockdown during March–May 2020). The high incidence and death rates of COVID-19 both in Switzerland and in the neighbouring countries during October-November 2020 and the spread of the new variants (especially B 1.1.7. in Switzerland before Christmas and B.1.351 in Tirol in January 2021), urged the Canton Grisons to launch a population-based mass testing campaign in order to intensify their mitigation strategy while maintaining cross-border socio-economical relationships.

Based on the experience acquired from a few rounds of pilot mass testing conducted in selected municipalities, a repetitive testing strategy for employees was developed for the entire Canton. In the first week of February 2021, 174 companies were recruited, with approximately 100 businesses being added every week since then. The decision to focus on the working force was taken to maximize the benefits by targeting individuals with high mobility and large network of contacts (such as hotel employees), and to enable business continuity by preventing outbreaks in professional networks.

The mass testing campaign relies on voluntary repetitive testing of unvaccinated employees, who are mostly tested once per week (twice per week in a few cases). If the test turns positive, employees are asked to self-isolate, while their work contacts are identified and offered daily testing for ten consecutive days. As long as they remain negative, they are allowed to continue to work on company’s premises with few restrictions (e.g. wearing mask and minimizing contacts when possible): they are asked to self-isolate only if their own test result is positive. This “test-and-release” protocol minimizes the number of people in quarantine and thereby limits possible burdens on the participating businesses. In addition, external contacts (e.g., family and friends) of the infected employee are informed to take precautionary measures and test according to the associated contact tracing program. Even though they are not included in the mass testing program, their risk of infection will be reduced by having a part of their contact network (the employees enrolled in the mass testing program) regularly tested and a beneficial secondary effect is expected for the general canton’s population.

The collected saliva samples are analyzed by the Reverse Transcription Polymerase Chain Reaction (RT-PCR) methods. The use of saliva samples ensures convenience of participants during the testing process. By quantifying the operative characteristics of the tests, a high specificity of 0.999873 (corresponds to 1 false positive in 7900 tests) and sensitivity close to nasopharyngeal swab RT-PCR were found^15^. Test processing is expedited by mixing the pools of 5 samples (5 to 1) by the automated platform in the laboratory. The results are communicated within 24 hours, resulting in a test-to-notice time of around 1 day (see *Supplementary materials, Study design and data acquisition*).

In total, 1022 businesses, operating on 1358 sites, joined the program from the beginning of February until the end of March, 2021. A total of 121,364 tests were conducted among 27,514 employees without symptoms. Positive test results were found in 215 cases (0.78%) (see *Supplementary materials, Data pre-processing*).

### Factors affecting evaluation and how to quantify

Quantifying the effect of such a repetitive mass testing program in businesses in a real-time setting is not straightforward.

On the one hand, estimates of the population-based indicators to quantify the effect size on the epidemic dynamics, such as the reproductive number, are confounded by the number of tests performed which was not constant over the considered period^16,17^.

On the other hand, the epidemiological situation changed over time. The proportion of the B.1.1.7 variant increased as a result of its higher transmissibility, whereas the population aged above 80 was being immunized during the period of our analysis, reducing the size of the susceptible population.

Finally, different business sectors (e.g. tourism, banking, construction, with different number of clients and cross-border employees) are present at varying proportions over time. This might significantly influence the effectiveness of the program, since companies with a higher number of customer contacts or cross-border employees are prone to a higher disease incursion.

For these reasons, instead of focusing on population-based epidemiological quantities in the Canton, here we limit ourselves to a finer-grain analysis: we evaluate the impact of testing on the evolution of the epidemics among participants. Therefore, we consider two groups of employees: the first group consists of the newly enrolled ones, the second group of the ones who have been enrolled in the program for a longer time and have therefore been tested repeatedly. The testing results of the newly enrolled participants are used as a proxy for the spread of infection among the population outside of the program (control group without testing). To account for the influence of different epidemiological conditions at the time of enrollment, which change over time, we divided the repeatedly tested employees into different cohorts according to the week during which they joined the program (i.e., we define week 1, week 2, and week 3 cohorts) (see *Supplemenraty materials, Cohort definition* ). Here, we analyze the first two months of this program until the Easter break period, for which the data is already consolidated.

## Results

### Test positivity rate and incidence rate ratio

**Figure 1:**
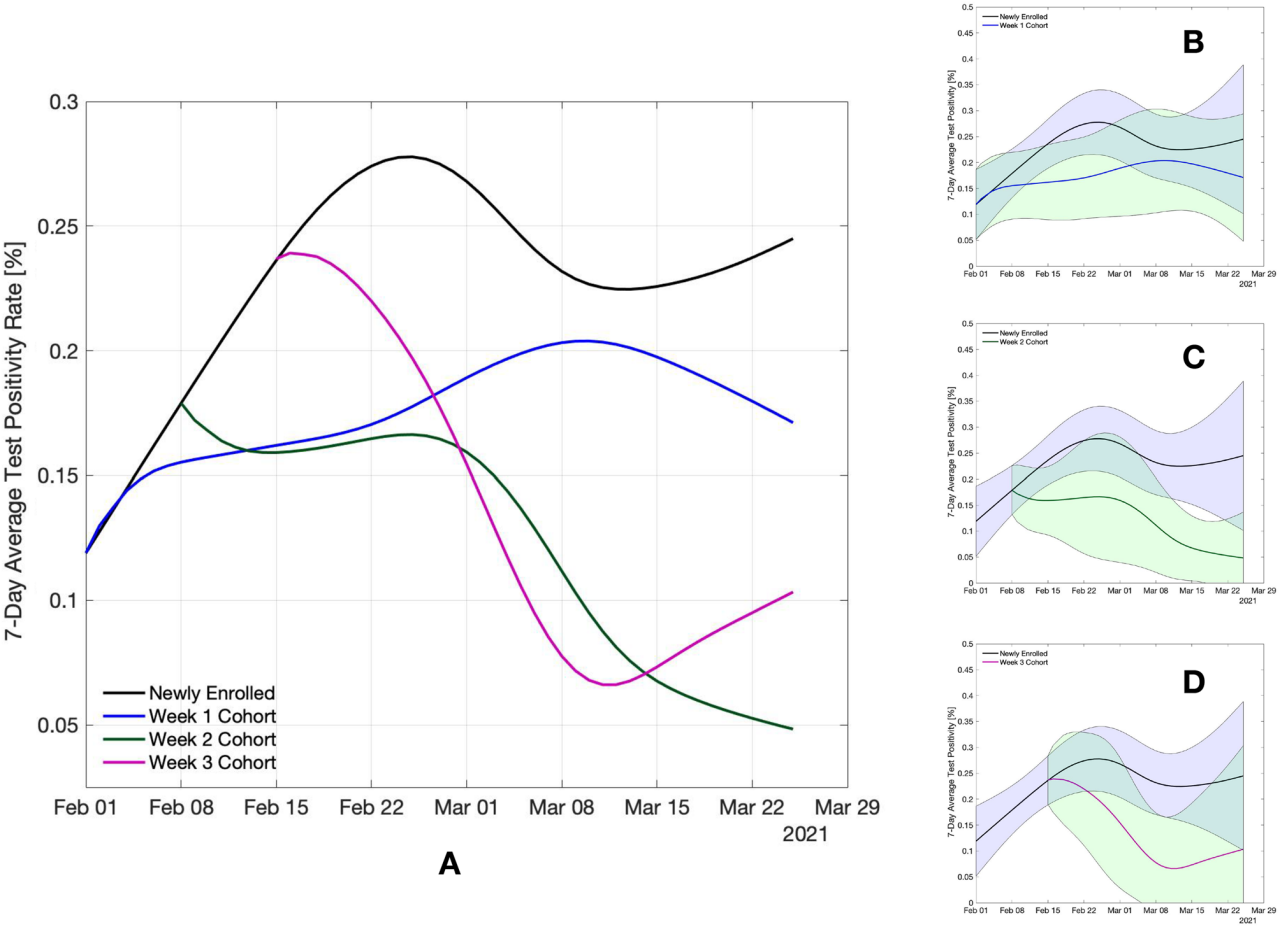
Evolution of TPR (%) for the newly enrolled population as well as other three cohorts considered. (**A**) LOESS fitted 7-day average TPR. (**B**–**D**) LOESS fitted 7-day average TPR overlaid by 95% CI computed via bootstrapping of the data set (see Fig 4 in *Supplementary materials* for non-smoothed 7-day TPR).

To infer the epidemiological changes among tested employees, we compute two complementary quantities: test positivity rate (TPR) and incidence rate ratio. The seven-day average TPR is calculated as the proportion of new positive cases to the total number of RT-PCR tests per seven days. Figure 1 shows the LOESS fit of the seven-day average TPR for the newly enrolled population (black line) along with the same quantity for repeatedly tested cohorts (blue, green and pink lines), (see *Supplementary materials, Overview of statistical methods*). We observe an increase in TPR among newly enrolled employees during the month of February, which is consistent with the increase in frequency of outbreaks in schools registered in this region, as well as with a concurrent expansion of the pandemic in several Swiss regions. The increase of TPR among the newly enrolled population results in quite different upstream epidemiological conditions for the different tested cohorts. Especially, the initial TPR value of the week 3 cohort is twice as high as the one corresponding to the week 1 cohort. Even though TPR was lower during enrollment of the week 1 cohort, this cohort shows a remarkably smaller and more uncertain reduction in TPR than the other two cohorts joining in later weeks.

The reduction of TPR over the period in which the cohort is repeatedly tested varies from 73% (over 7 weeks) to 56% (over 6 weeks), for week 2 and week 3 cohorts, respectively. The week 1 cohort shows a 44% increase over the entire eight-week period. Notice that during the same eight-week period, TPR of the newly enrolled population almost doubles (reaching a peak of 0.28% in the fourth week of the program). In general, all three repeatedly tested cohorts of employees exhibit evident reductions in TPR when compared to the newly enrolled population. Yet given the relatively important uncertainty resulting from our small sample size, the critical question is if the reductions in TPR translate into a statistically significant drop in the incidence rate of the tested cohorts.

As an integral measure of the reduction in the number of infections over the entire period, we calculate the incidence rate associated with the seven-day average TPR by normalizing the number of new positive cases by the person-time tested (see *Supplementary materials, Overview of statistical methods*). The ratio between the incidence rate of each cohort with respect to the newly enrolled population is provided in Table 1. The p-values reported in the table, estimated by mid-p exact method, correspond to the test that the incidence rate in the considered cohort has a lower value than the one in the newly enrolled population, hence testing is effective in reducing the number of infections. The estimated reduction in the incidence rate is 18% for the week 1 cohort, 47% for the week 2 cohort and 50% for the week 3 cohort (see Table 1). For the week 2 and week 3 cohorts, we find a statistically significant reduction of the incidence rate achieved by repetitive testing (p-values 0.03 and 0.04, respectively).

The milder reduction in TPR and incidence rate of the week 1 cohort can partly be explained by a higher exposure to exogenous disease incursion due to the higher representation of the tourism sector and cross-border employees in week 1 cohort in comparison to week 2 and week 3 cohorts (for more details see also *Supplementary materials, Effect of tourism and cross-border commuters* ).

**Table 1:** Ratio between the incidence rate of each repeatedly tested cohort with respect to the newly enrolled population.

Starting week of cohort	Estimate	p-value	95% CI
Week 1	0.82	0.36	(0.53,1.25)
Week 2	0.53	0.03	(0.28, 0.95)
Week 3	0.50	0.04	(0.24, 0.96)

### Tourist season and exogenous contacts

**Figure 2:**
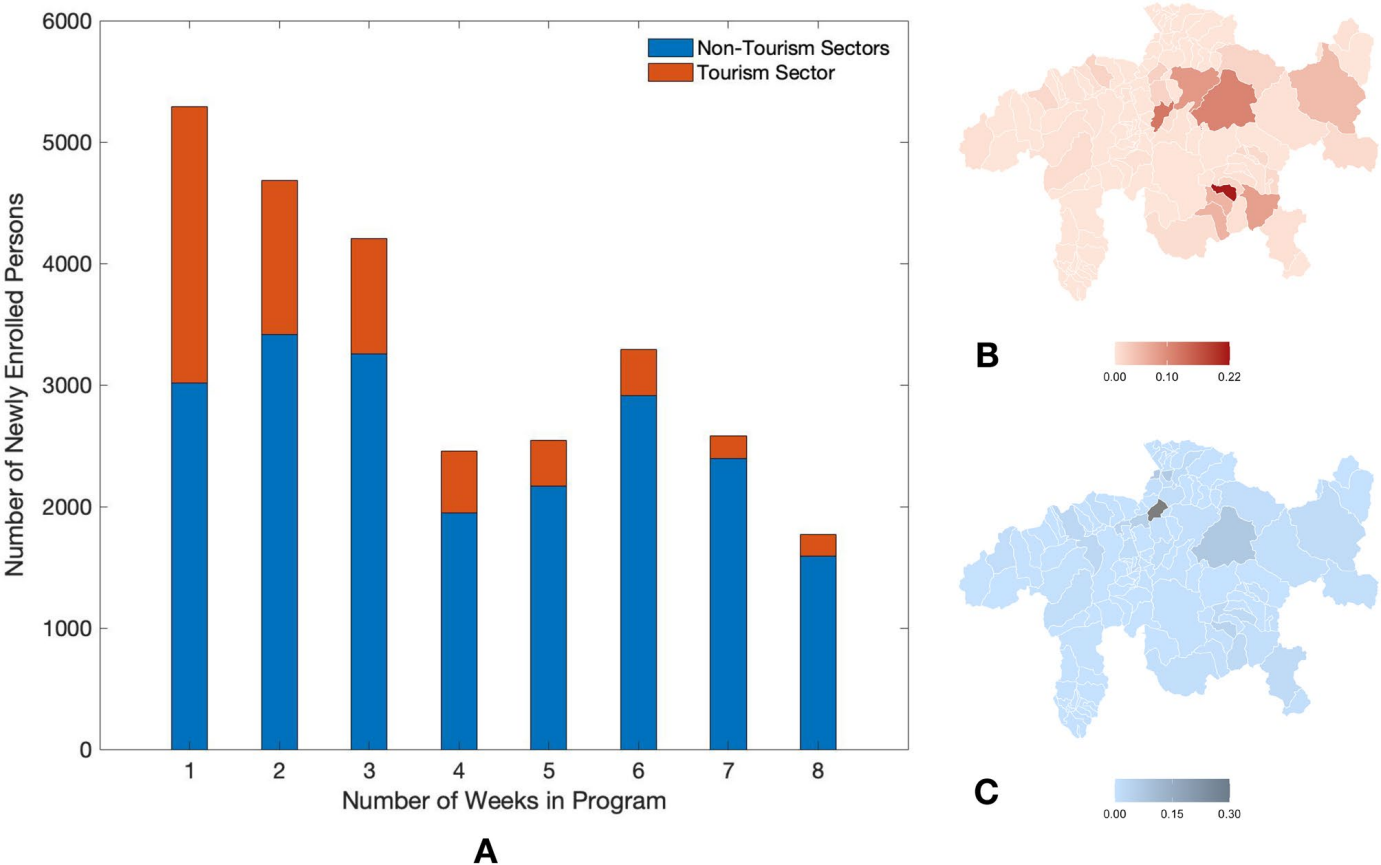
Enrollment and geographic distribution. (**A**) Number of newly enrolled employees versus the number of weeks since initiation of the program. (**B**,**C**) The geographic distribution of work-places of program participants in tourism sector and non-tourism sectors, respectively (see Fig. 3 in *Supplementary materials* for more details). The maps were generated by the R package RSwissMaps http://cran.nexr.com/web/packages/RSwissMaps/index.html.

Tourism industry is one of the major economical resources of the Canton Grisons. During the past winter season (December 2020 to March 2021), around 200,000 tourists resulted in an additional exogenous infection source in the Canton. Around a quarter (26%) of all tests were carried out among people employed in the tourism sector. Remarkably, this business sector accounted for almost half (48%) of all positive tests, which results in a frequency of positive cases that is close to two times larger than in other business sectors.

Figure 2 indicates that approximately 45% of the participants in the first week cohort work in the tourism sector. This fraction reduces to 20-30% for the cohorts of weeks 2 and 3, and keeps decreasing further afterwards. The businesses participating from the tourism sector are broadly distributed, representing a comparatively larger fraction of businesses in the regions of St. Moritz, Davos, Pontresina and Arosa. Conversely, the other business sectors are more concentrated in Chur and to a less degree in Davos. In the first week cohort we observe a prevalence of individuals employed in companies located in Davos, St. Moritz, and Pontresina, as well as a noticeable fraction of individuals from peripheral regions with shared borders (e.g., Bregaglia). Instead, the cohorts of week 2 and week 3 are characterized by a higher representation of the region of Chur, which hosts offices and factories, in addition to the touristic region of St. Moritz which is also present in week 1 cohort (see *Supplementary materials, Cohort definition*).

The number of tourists peaked around December and February, and started declining towards the middle of March. This variation in the number of tourists could explain the increase of TPR among the newly enrolled individuals (control group without testing) during the month of February (peaking around the fourth week) and the decline observed afterwards. The tourists and the large proportion of foreign workforce employed in the tourism industry are a potential source of exogenous disease incursion and can noticeably contribute to the incidence of COVID-19 in the Canton Grisons. The fluctuation in the proportion of businesses from the tourism sector in the three cohorts is a potential source of confounding effect in our analysis.

In particular, the week 1 cohort bears the highest proportion of the tourism work force and shows the least reduction in TPR. This might be explained by two effects. First, the employees who were mostly in contact with visiting tourists were subject to a higher disease transmissibility, resulting from a larger number of contacts with untested individuals (as tourists are not part of the program) and possibly coming from regions with higher prevalence. Second, many employees in this sector were crossing borders on a daily or weekly basis, hence their risk of exposure to infected individuals might differ from that of employees in non-tourism sectors, which mostly remained within the Canton during the time of our study. These hypotheses are supported by an analysis performed on a reduced data set from which a few touristic and near-border municipalities have been excluded. The reevaluated reduction in the incidence rate almost doubles from 18% to 34% for week 1 cohort, and increases from 48% to 51% for week 2 cohort, and from 43% to 61% for week 3 cohort (see Table 4 and *Supplementary material, Effect of tourism and cross border commuters* for the details).

## Discussion

The question whether repetitive mass testing can play a key role in response to COVID-19 pandemic, has important health, economical and social implications. The data collected from business testing program of the Canton Grisons provide the first empirical evidence that repetitive mass testing is indeed effective to prevent spread of the epidemic. Even though the repetitive testing campaign for COVID-19 mitigation on such a population scale and over such an extended period of time is unprecedented, our sample size remains relatively small: about 200 positive tests resulting from repetitive testing of around 27,000 individuals over eight weeks. This results in a considerable uncertainty in our analysis. Nevertheless the reduction in the incidence rate that we observed is statistically significant, especially among the groups of employees with lower exposure to exogenous disease incursions (e.g., individuals not employed in the tourism sector nor living in touristic/peripheral regions). This is encouraging as it shows that intensifying repetitive testing can be a viable complement to the existing non-pharmaceutical intervention strategies to reach epidemic control.

Several features contributed to the success of our testing program. The use of saliva based RT-PCR tests, with no nasal swab required, has certainly fostered a high participation rate and consistent repetitive testing: the program uptake among employees was 74% during February-March 2021, with an estimated drop-out by the second month lower than 10%. Furthermore, the high specificity of the employed RT-PCR tests assures that only a few false positive cases can be expected; this is important to keep testing and self-isolation effective also when the prevalence of the tested population is relatively low. Finally, the efficiency of the test analysis was improved by automated pooling of the samples in the laboratory, which allowed us to scale up the size of the tested population for a given testing capacity and to keep the test-to-notice time short.

To define the objectives and plan a successful repetitive testing concept, it is important to carefully consider the interaction within and between the tested and untested sub-populations, as their dynamic is far from being homogeneous. On the one hand, the repeatedly tested individuals actively contribute to curb the virus spread: as the tested cohort itself benefits from a reduced incidence, their untested contacts are also exposed to a lower risk of infection. On the other hand, interactions of tested cohort with individuals outside of the program expose the former to the transmission risk originated from the latter, reducing the effectiveness of testing.

The presented study is prone to certain limitations. Most importantly, our results rest on the assumption that there was no significant variation of confounding factors (e.g. vaccination status, age and socio-economic background) among the different cohorts that have been considered. As the time window of the analysis predated the general public vaccination campaign, the analyzed data concerned people who were not vaccinated. Moreover, due to the lack of detailed data on the participants, we considered the business sector as a proxy for both age-distribution and socio-economic status of the tested work forces. Our results hint that the impact of the testing program in cohorts with similar mix of business sectors tend to be alike, which supports the choice of the business sector as the relevant proxy.

Our analysis supports that a higher number of active participants per company site tended to correlate with smaller incidence rates, hence confirming the requirement of achieving a sufficiently large uptake among employees (*Supplementary materials, Overview of statistical methods*). Also, a higher number of tourists or cross-border employees from regions not covered by the testing program represented a potential source of disease incursion and were associated with a lower reduction of the incidence rate. In order to more efficiently control the epidemic, therefore it is important to adapt the testing program according to the varying level of exposure to the exogenous sources of infection. This can be achieved, for instance, by a larger uptake and a more frequent testing among individuals with an extended network of contacts outside of the program.

If carefully designed, repetitive mass testing programs can complement other public health measures, limiting the socio-economical tolls resulting from extreme interventions. In the current stage of COVID-19 pandemic, resorting to repetitive mass testing programs remains a valuable measure, for instance, in societal branches for which vaccination is not yet possible or in which outbreaks due to exposure to new mutants is highly probable (e.g., at schools or in the tourism sector). This requires a coordinated engagement of policy makers, communities, and the public to adopt carefully designed testing programs that could be sustained over an extended period of time.

## Supplementary Information

Supplementary Information.

## Data Availability

The source data set is available on https://www.gr.ch/DE/institutionen/verwaltung/djsg/ga/coronavirus/info/medien/Seiten/Medien.aspx. The analysis was implemented with R. The corresponding codes are available upon request from the corresponding author.
