# Supplementary material for: Results from Canton Grisons of Switzerland suggest repetitive testing reduces SARS-CoV-2 incidence (February–March 2021)
Source: Sci Rep. 2022 Nov 14;12:19538. doi: 10.1038/s41598-022-23986-0 (PMC9663184; doi:10.1038/s41598-022-23986-0)
Supplement: Supplementary file 1 — Supplementary Information. [file 41598_2022_23986_MOESM1_ESM.pdf]

Supplementary materials

Methods

Figs. 3 to 4

Tables 2 to 4

Methods

Study design and data acquisition

After the company signs up for the testing program, the employees register to the IT system and the validity of their data entry is confirmed. Saliva samples are collected by rinsing the oral cavity with 2 ml reagent grade water for one minute. Program participants are instructed not to eat, drink, smoke or brush teeth 1 hour before sample collection. The fluid is then collected by means of a plastic funnel into a dedicated CE-marked sterile sample tube containing crystalline guanidine thiocyanate in order to inactivate the sample and stabilize viral RNA. We determined stability of RNA-levels at room temperature for 5 days. Testing vessels are delivered by the Swiss post. For the collection of the samples, the employer delivers the samples to predefined collecting points from where the Swiss post together with the regional train, transport the samples to the lab.

Pools of 5 samples received at the same day in the laboratory are mixed in a fully automated platform preventing contamination in the pipetting procedure. For each single program participant an additional separate sample is produced and stored in a sample archive. RNA of the pool sample is purified by means of an automated extraction platform. The SARS-CoV-2 specific RNA is then amplified by means of a commercial test kit run on a QuantStudio 5 instrument. This assay tests for presence of 3 different genes of the SARS-CoV-2 virus (N-gene, ORF1ab-gene, S-gene). Negative and positive control materials are tested with each run. When a pooled sample tested positive or ambiguous, the stored samples of the pool participants are analyzed at an individual level, in order to identify individuals positive for SARS-CoV-2. The results are communicated within 24 hours upon submission of the samples.

Positively identified individuals are asked to self-isolate for ten days. The quarantine of the work contacts of the positive cases is waved and replaced by daily testing for maximum of ten days; the work contacts of the positive case are asked to self-isolate only in case their test results turns out positive. Test results from the laboratory are entered into the IT platform by hand and the results are expressed in MS Excel spreadsheets. The pseudonymized data set includes unique identifiers for the company site and the tested employee, besides the testing date, the test result, the municipality of the company and the business sector.

Data pre-processing

From 146'823 entries related to company testing in February and March 2021, 25'449 (17%) entries did not have a test result. This was typically the case when an employee gets a testing voucher but does not take the test. We remove the records without test results, and analyze only the curated data set.

In the case of multiple tests per seven-day time window, only one test per person is counted. The result of the test is considered positive if any of the tests taken in the seven-day time window gives a positive result.

The contacts of positive cases comprise a high-prevalence sub-population, thus the chance that they are identified as positive is higher than for a randomly chosen person from the same cohort (as contacts are tested daily). On the other hand, excluding the contact testing records from the analysis would make the number of positive cases in repeatedly tested cohorts biased towards lower values (as the contacts of positive cases would not be counted). Therefore we make a conservative choice and do not differentiate between the test results of the contact testing program from the rest. We expect that an ideal random testing program would reach a stronger

effect compared to the results of our analysis.

In total, 266 tests rendered positive results, yet 51 are repeated positive tests (19% of all positive tests). This is due to three main reasons:

1. The delay in the test-to-notice process for daily contact testing can lead to a scenario where a person is tested on the following day while still uninformed about positive result of the past day. As a result, two positive tests of the same person in two consecutive days would be recorded in the data set.

2. After recovery, a person might still show up positive in the PCR test.

3. In a few cases, the positive tested employee does not adhere to the self-isolation rule and repeats the test.

In our analysis, which covers a period of two months, we count a positive test result only once per person.

Cohort definition

We define the following sub-populations of test results.

1. Newly enrolled population: A test is counted in the newly enrolled statistics if the person hasn't been previously tested in our testing campaign, and if the company site has not been in the program for more than two weeks prior to the test date. The latter condition is introduced to exclude (to some extent) the effect of community protection due to testing, which might occur among those employees of a company that have started to participate later.

2. Week 1-3 cohorts: These cohorts are composed of employees that enter the program during the same week (numbered sequentially from the start of the program) and named

week 1, week 2, and week 3 cohorts. During its first seven days each cohort coincides with the newly enrolled population. We only count an individual in the cohort if at least 5 persons from the same company site participate consistently (i.e., they are tested every week). In other words, we only keep those individuals in the cohort, whose companies sites remain active in the program. Notice that the results are robust with respect to the adopted minimum number of active participants per company site, henceforth called ”unit cohort size” (illustrated in Tables 2 and 3).

Figure 3 shows the geographical distribution of the work places of newly enrolled employees. The share of touristic and near-border regions is noticeably higher in the week 1 cohort than in the cohorts of the two subsequent weeks.

The program participation rate during February-March 2021 has been 74%; 9.9% of employees who tested at least once during February, submitted no test during March, suggesting an estimated drop-out rate of 10% per month.

Overview of statistical methods

The daily data are too scarce and largely affected by the weekly periodicity, particularly visible in the consistently lower number of tests during the weekend. Therefore, we apply a moving average with a seven-day time window (forward in time). The following two analyses are then performed.

1. Test Positivity Rate (TPR) : To compute the seven-day average TPR at a given date in a certain sub-population, the total number of discovered positive cases is divided by the number of tests in the seven-day window. Only one test per person is counted per considered period. If τ is the time (day), let $\bar{I}(\tau)$ and $\bar{N}(\tau)$ be the total number of new positive cases detected and the number of tested persons during the period $[\tau, \tau + 6]$, respectively.

Therefore the seven-day-average TPR is given by TPR$(\tau) = \bar{I}(\tau)/\bar{N}(\tau)$. The histograms in Fig. 4 (A), (B) and (C) show the resulting TPR for newly enrolled employees side by side with the week 1,2 and 3 cohorts, respectively.

For smoothing and uncertainty quantification of time series data, we follow an approach similar to the one discussed in [18, 19]. The seven-day average TPR is smoothed using a first order local polynomial regression (LOESS) algorithm with bandwidth 0.65 [20]. To quantify the uncertainty band, we apply bootstrapping prior to the computation of the seven-day average TPR and its corresponding LOESS fit. A resampled record is obtained by sampling entries from the original data set under uniform probability and replacing them in the data set [21, 22]. Thus the obtained resampled data set has a length similar to the original one. We generated 500 resampled data sets, and applied normal distribution for quantifying uncertainty of the estimated statistics. To ensure that the starting points of week 1,2 and 3 cohorts and their uncertainties lie on the ones obtained from the newly enrolled population after smoothing, the LOESS weights are locally adjusted and the neighbourhood of the intersection points are smoothed again by moving averaging. The LOESS fitted TPR and its 95% CI are shown in Fig. 1 (B), (C) and (D) for week 1,2 and 3 cohorts, respectively, besides the newly enrolled population.

2. Incidence Rate Ratio: In order to extract information with a lower uncertainty than the TPR time series, we complement the analysis by calculating the incidence rate as an integral measure over the entire considered period. Accordingly, the following analysis would not rely on smoothing the seven-day average time series, and thus the results are independent of the LOESS fit. We compute the incidence rate for each sub-population as proportion of the total number of new positive cases to the person-time, i.e. each person is counted for the number of weeks being tested in the sub-population. Therefore, the

adopted incidence rate is an approximation of the area below the TPR curve (normalized by time). In order to avoid complications in our incidence rate estimations, we assume unity sensitivity and specificity of the tests. Furthermore, the time lag between infection and detection via the test is neglected. By binning the data of each cohort into weeks, we estimate the incidence rate of cohort 1 from week 2 till 8, cohort 2 from week 3 till 8, and cohort 3 from week 4 till 8. The incidence rates are compared with the incidence rate of the newly enrolled population for the same time period.

Consider IR_c^j to be the incidence rate for the repeatedly tested cohort that joins the program at week $j \in \{1, 2, 3\}$. Let $N^j(i)$ and $I^j(i)$ be the number of tested people and number of new positive cases, respectively, at week i in the cohort j. Therefore the incidence rate for a cohort that starts at week j is

$$\mathrm{IR}_c^j = \frac{\sum_{i=j+1}^{8} I^j(i)}{\sum_{i=j+1}^{8} N^j(i)} . \tag{1}$$

Accordingly, for the newly enrolled incidence rate IR_0 we have

$$\mathrm{IR}_0(n_0) = \frac{\sum_{i=n_0}^{8} I_0(i)}{\sum_{i=n_0}^{8} N_0(i)} , \tag{2}$$

where $N_0(i)$ and $I_0(i)$ are the number of tested people and discovered positive cases, respectively, at week i among the newly enrolled population. In order to compare the incidence rate of each cohort with the newly enrolled population for a similar time interval, we set n_0 to 2, 3 and 4, for comparing IR_0 to $\mathrm{IR}_c^{j=1}$,$\mathrm{IR}_c^{j=2}$ and $\mathrm{IR}_c^{j=3}$, respectively. The corresponding reduction in the incidence rate for a cohort starting at week j then follows

$$C^j = 1 - \frac{\mathrm{IR}_c^j}{\mathrm{IR}_0(n_0 = j + 1)} . \tag{3}$$

The reduction in the incidence rate is computed from the incidence rate ratio estimated by the package Epitools [23]. The confidence intervals together with p-values are computed

using mid-p exact method [23, 24]. The results are provided in Table 1. Furthermore, we repeat the evaluation of the incidence rate ratio by changing the minimum number of consistent participants per company site. The results are given in Table 2 for unit cohort size of 3, and in Table 3 for unit cohort size of 7. While the estimates do not vary much with respect to the unit cohort size, we observe that larger unit cohort sizes tend to correlate with smaller incidence rate ratios, and thus stronger reduction in the incidence rate. For example in the week 1 cohort, we find that the reduction increases from 16% to 26%, once the unit cohort size grows from 3 to 7. This is in line with the expectation that more effective reduction in the incidence rate would be achieved in companies, where larger pools of employees participate in the program.

Effect of tourism and cross-border Commuters

To better understand the disparity in TPR and incidence rate reduction of different cohorts, we analyze the effects of tourism-sector employees and cross-border commuters. While it is possible to exclude from the data set the employees defined as working in the tourism sector and reevaluate the effect of repetitive testing in the filtered data set, it may not provide a correct evaluation of the effects of visitors and commuters. Indeed, not all business branches that deal with tourist clients are categorized as tourism sector (e.g., employees that provide various services in train stations interact often with tourists but are not counted as being employed in the tourism sector). Moreover, our cohorts are not isolated and interact with large portions of the population outside of the program. Also, due to the limited amount of data (resulting in wide uncertainty bounds, Fig. 1), removing a large portion of the data can be misleading. It may be more appropriate to take into account tourist-related infections by performing a regional analysis of the data rather than separating the different business sectors. Therefore, we decided to exclude a few touristic and near-border municipalities from the data set.

In the week 1 cohort we identify three regions with the highest number of employees working in the tourism sector: Davos, Pontresina and Vaz/Obervaz. We remove from the data set these municipalities as well as two peripheral regions Landquart and Bregaglia, which are more represented in the week 1 cohort than in the other two. In the filtered data set we observe that the share of tourism sector is shrank from 43% to 34% in week 1 cohort, from 27% to 26% in week 2 cohort, and from 23% to 22% in week 3 cohort. The incidence rate ratio of each cohort with respect to the newly enrolled one is re-evaluated and reported in Table 4. The incidence rate reduction almost doubles from 18% to 34% for week 1 cohort, and increases from 48% to 51% for week 2 cohort, and from 43% to 61% for week 3 cohort. This supports the hypothesis that the relatively small reduction of TPR of the week 1 cohort in the original data set can be explained by an over representation of regions with higher rates of tourists and cross-border commuters.

Ethics declarations

All methods were carried out in accordance with relevant guidelines and regulations. Informed consent was obtained from all participants. The study was reviewed by the Cantonal Research Ethics Commission of Zurich Switzerland (KEK-ZH), and obtaining ethical approval was waved.

Additional Information

Competing Interests

L.R. and M.R. are key shareholders in Risch laboratories, which provided the laboratory testing. Others declare no potential conflict of interest.

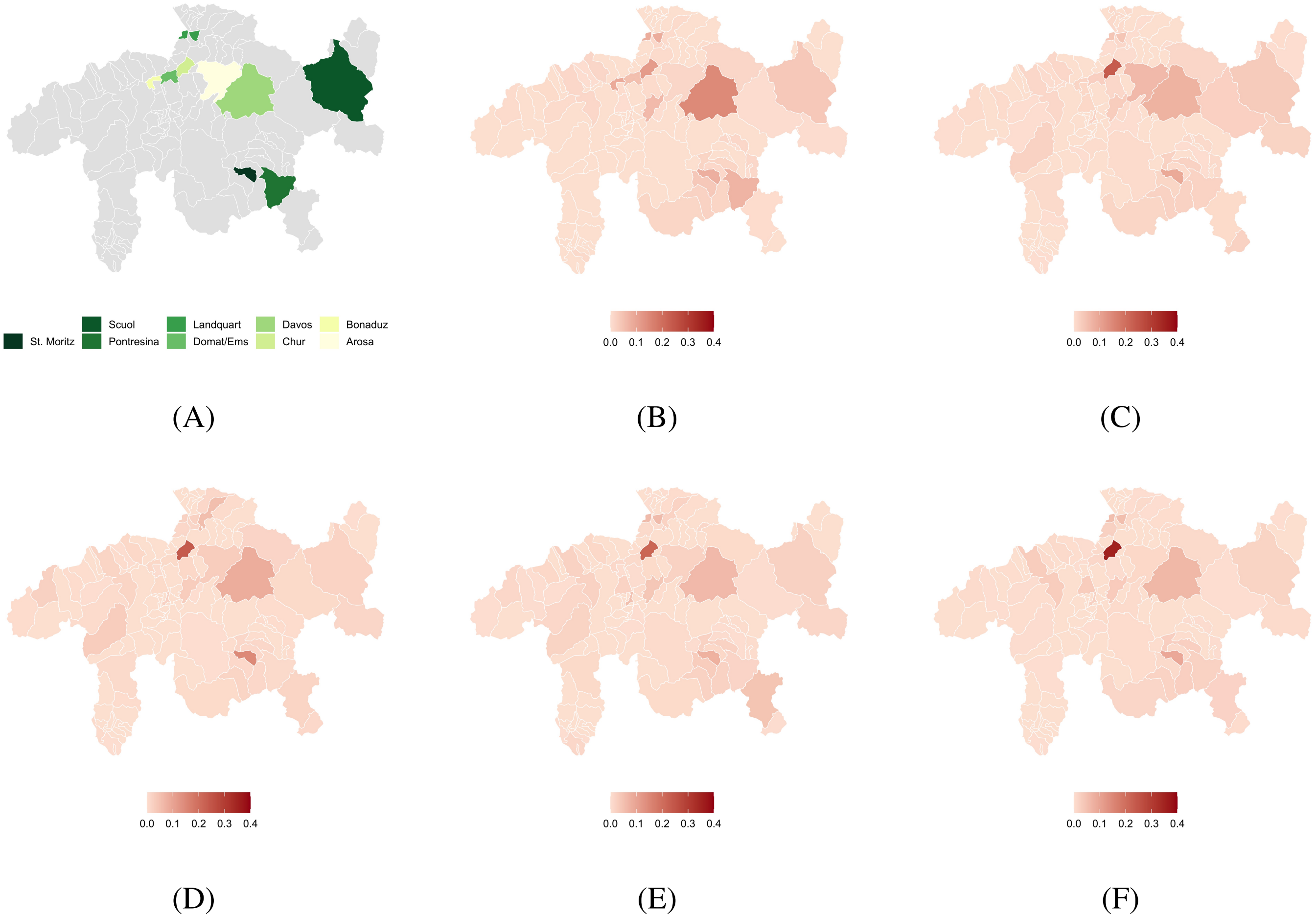

Figure 3: Geographical distribution of the participating businesses. Enrollments from week 1 to week 5 correspond to panels (B) to (F) , respectively. The distribution of the work place location of newly enrolled employees is color-coded for each week. Panel (A) provides names of few municipalities in the Canton Grisons. The maps were generated by the R package RSwissMaps http://cran.nexr.com/web/packages/RSwissMaps/index.html.

Table 2: Ratio between the incidence rate of each repeatedly tested cohort with respect to the newly enrolled population, for unit cohort size of 3

Starting week of cohort	Estimate	p-value	95% CI
week 1	0.84	0.42	(0.55,1.28)
week 2	0.48	0.01	(0.26,0.85)
week 3	0.54	0.05	(0.27,1)

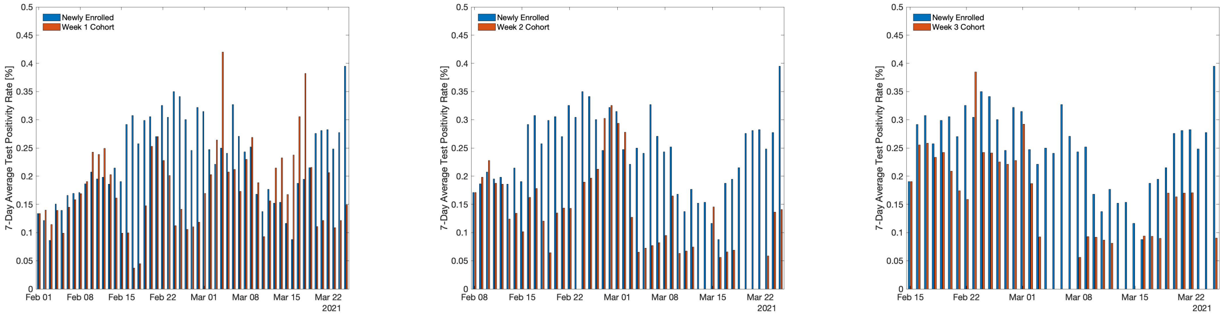

(A) (B) (C)

Figure 4: 7-day average TPR (%). Panels (A), (B) and (C) show the TPR for newly enrolled employees side by side with the week 1,2 and 3 cohorts, respectively.

Table 3: Ratio between the incidence rate of each repeatedly tested cohort with respect to the newly enrolled population, for unit cohort size of 3

Starting week of cohort	Estimate	p-value	95% CI
week 1	0.74	0.18	(0.46,1.15)
week 2	0.49	0.02	(0.27,0.91)
week 3	0.47	0.04	(0.21, 0.95)

Table 4: Ratio between the incidence rate of each repeatedly tested cohort with respect to the newly enrolled population, excluding touristic and peripheral regions

Starting week of cohort	Estimate	p-value	95% CI
week 1	0.66	0.14	(0.36,1.15)
week 2	0.49	0.03	(0.24,0.92)
week 3	0.39	0.01	(0.16,0.83)
